# A review of therapeutic effects of mesenchymal stem cell secretions and induction of secretory modification by different culture methods

**DOI:** 10.1186/s12967-014-0260-8

**Published:** 2014-10-11

**Authors:** Marialaura Madrigal, Kosagisharaf S Rao, Neil H Riordan

**Affiliations:** Department of Biotechnology, Acharya Nagarjuna University, Guntur, India; INDICASAT-AIP, City of Knowledge, Republic of Panama; MediStem Panama Inc., City of Knowledge, Republic of Panama

**Keywords:** MSC, Hypoxia, Inflammation, Spheroids, VEGF, PGE2, TSG-6, Cell therapy, Conditioned media

## Abstract

The mesenchymal stem cell (MSC) is being broadly studied in clinical trials. Contrary to the early paradigm of cell replacement and differentiation as a therapeutic mechanism of action, evidence is mounting that the secretions of the cells are responsible for their therapeutic effects. These secretions include molecules and extracellular vesicles that have both local and distant effects. This review summarizes the up- and down-regulation of MSC anti-inflammatory, immune modulating, anti-tumor, and regenerative secretions resulting from different stimuli including: a) hypoxia, which increases the production of growth factors and anti-inflammatory molecules; b) pro-inflammatory stimuli that induce the secretion of immune modulating and anti-inflammatory factors; and c) 3 dimensional growth which up regulates the production of anti-cancer factors and anti-inflammatory molecules compared to monolayer culture. Finally we review in detail the most important factors present in conditioned medium of MSC that can be considered protagonists of MSC physiological effects including HGF, TGF-b, VEGF, TSG-6, PGE2 and galectins 1, and 9. We conclude that there is potential for the development of acellular therapeutic interventions for autoimmune, inflammatory, and malignant diseases and tissue regeneration from cellular secretions derived from MSCs cultured under the appropriate conditions.

## Introduction

Mesenchymal stem cells (MSCs) are classically defined as adherent, non-hematopoietic cells expressing the surface markers CD90, CD105, and CD73, and lacking the expression of CD14, CD34, and CD45. The cells also have the capacity to differentiate into adipocytes, chondrocytes, and osteocytes *in vitro* after treatment with differentiation inducing agents [1]. Although early studies in the late 1960s initially identified MSCs in the bone marrow [2], more recent studies have reported these cells can be purified from various tissues such as adipose [3], heart [4], Wharton’s jelly [5], dental pulp [6] peripheral blood [7], cord blood [8], and more recently menstrual blood [9-11] and chorionic villi [12]. Studies of bone marrow showed that although MSC are the primary cell type that overgrow *in vitro* cultures, *in vivo* MSC are found at a low ratio compared to other bone marrow mononuclear cells, specifically, 1:10,000 to 1:100,000 [13]. The physiological role of MSC still remains to be fully elucidated, with one hypothesis being that bone marrow MSC act as precursors for stromal cells that make up the hematopoietic stem cell microenvironment [14-16].

The first clinical use of MSCs was to accelerate hematopoietic recovery after bone marrow ablation in the context of post chemotherapy hematopoietic stem cell transplant. Lazarus *et al*. report of the use of autologous, 1–50 × 10^6^ cells *in vitro* expanded, “mesenchymal progenitor cells” to treat 15 patients suffering from hematological malignancies in remission and treatment showed no treatment-associated adverse effects [17]. In a subsequent study by the same group, MSC treatment accelerated hematopoietic reconstitution in 28 breast cancer patients who received high dose chemotherapy with no reported treatment associated adverse effects. The authors noted that leukocytic and thrombocytic reconstitution occurred at an accelerated rate as compared to historical controls [18]. In addition to feasibility, these studies importantly established techniques for *ex vivo* expansion and administration.

Demonstration of clinical feasibility and multiple animal models providing rationale for therapeutic efficacy of MSCs in non-hematopoietic indications [19-26], gave rise to a series of clinical trials of MSCs in a wide range of major diseases including stroke [27-30], heart failure [31,32], COPD [33] and liver failure [34]. Rare diseases treated with MSCs such as osteogenesis imperfecta [35], Hurler syndrome [36], and Duchenne Muscular Dystrophy [37] have also been reported.

The ability to generate clinically significant numbers of well-defined MSCs starting with small clinical samples, feasible administration without the need for haplotype matching, and excellent safety profile of the cells has resulted in a broad interest in the clinical use of MSCs. 402 clinical trials testing MSC are currently listed on the international registry www.clinicaltrials.gov. While some trials have demonstrated efficacy of MSC, full elucidation of mechanisms of action is lacking. Initial studies demonstrated the ability of certain MSC types to differentiate into functional tissues that is compromised as a result of the underlying pathological. In spite of the capacity of MSCs to differentiate, evidence is mounting that much of the disease-modulating activity of MSCs is due to products secreted by the cells.

This paracrine effect was first observed in heart disease murine models, in which it was found that bone marrow (BM) MSCs injected into infarcted hearts did not differentiate into cardiomyocytes under physiological in vivo conditions [38]. After intravenous injection the majority of administered MSCs lodge in lungs and liver with only a small minority entering the tissue of pathology [39]. Gnecchi hypothesized that clinical effects of MSCs are not due to cell differentiation, after observing re-establishment of cardiac function and prevention of ventricular remodeling in fewer than 72 hours post injection [40]. The same group went on to show that MSC conditioned medium alone enhanced recovery of ischemic cardiomyocytes *in vitro* [41]. Similarly Lee and colleagues showed an anti-inflammatory effect and cardiac infarct size reduction; in spite of the fact that the majority of the intravenously infused MSCs (human MSCs in mouse model) were found as emboli in lungs and few cells migrated to other tissues including the infarcted heart [42]. Shabbir *et al*. demonstrated increased fractional shortening, and capillary and myocyte density, as well as attenuated myocyte apoptosis and fibrosis; in a hamster model of heart failure, in which MSCs were injected intramuscularly. They also showed that intramuscular injection of cell free MSC conditioned medium similarly rescued the failing heart in the same model [43].

Another example of what could be considered purely the effect of the MSC secretion was demonstrated in a rat model of complete transection of the spinal cord in which the termini of the cut cord were covered with fibrin glue containing human umbilical cord MSCs. The intervention improved the locomotion and resulted in regeneration of the spinal cord. It was found that human MSC antigen did not overlap with the staining for neurons, oligodendrocytes or astrocytes, demonstrating that there was a mechanism other than MSC differentiation involved in the spine cord lesion recovery [44]. Recently, Song *et al*., used a rat model of overactive bladder and demonstrated that MSC hardly engraft into damaged bladders, but increased stem cell gene expression suggesting an MSC paracrine effect is related to unleashing/mobilizing primitive progenitor cells as a possible mechanism for the long-term/stable therapeutic efficacy of MSCs [45].

The above-mentioned studies suggest that MSC efficacy may be mediated primarily by secreted factors. Identification of secreted factors will result in a better understanding of MSC therapeutic activity, which would allow not only for generation of MSCs optimized for efficacy for a potential target, but also the possibility of administering secreted factors that are naturally or synthetically generated as an alternative to use of live cells. There are a multitude of culture conditions that allow MSC to produce differing sets of trophic factors under biological need to be explored. In this paper, we will review MSC secreted factors including molecules and extracellular vesicles (exosomes and microvesicles) and culture conditions which can enhance the *in vitro* production of those secreted factors.

### MSC therapeutic activity is stimulated by physiological need

MSCs in standard monolayer culture secrete cytokines, micro RNA (miRNA), exosomes and microvesicles as a matter of course. The concept that MSC act as “repair cells” of the body would imply that MSC do not only constitutively secrete regenerative factors, but also produce some factors in response to stimuli. Responsive production and secretion is an experimental reality. Hypoxic preconditioning, addition of an inflammatory stimuli, and growing cells in spheres or tri-dimensional scaffolds have all been shown to modulate the production and excretion of different potential therapeutic factors. A summary of the effects of culture conditions on up-regulation of molecular secretion by MSCs and the up- and down-regulation of effects of MSCs and conditioned medium can be found in Table 1.

**Table 1:** **Summary of MSC secreted factors induced by Hypoxia, Inflammatory Stimuli and** ***3-*** **dimensional culture conditions and their effect on other cells**

**Molecule of interest**	**Hypoxia preconditioning** **(** **1** **-** **2%** **O**_**2**_ **)**	**Inflammatory stimuli** **(** **INF** **-** **γ,** **TNF** **-** **α,** **LPS** **)**	**3D culture configuration** **(** **microcarriers,** **microspheres** **)**
**FGF**	↑		
**VEGF**	↑	↑	
**IGF**	↑		
**HGF**	↑	↑	
**IDO**	↑	↑	
**Oct 4**	↑		
**Rex 1**	↑		
**TGF-** **β**		↑	
**PGE2**		↑	↑
**BMP2**		↑	
**Factor H**		↑	
**Gal** **-** **9**		↑	
**TSG** **-** **6**			↑
**STC** **-** **1**			↑
**CXCR4**			↑
**TRAIL**			↑
**IL** **-** **24**			↑
**CD82**			↑
**Secretion of microvesicles/** **exosomes**		↑	
**MSC or MSC** **–** **CM effect on other cells**			
**CXCL2**			↓
**TNF** **-** **α**			↓
**IL** **-** **6**			↓
**IL12p40**			↓
**IL23**			↓
**T** **-** **cell proliferation**	↓	↓	
**CD31** **+**	↑		
**αSMA** **+** **desmin** **+**	↑		

↑ = upregulated or stimulated; ↓= down-regulated or non-stimulated.

#### MSC stimulation by hypoxia

One of the most common elements of tissue injury is the presence of hypoxia. Interstitial damage is often associated with activation of the coagulation cascade, resulting in areas of hypoxia. It is known that reduction in oxygen tension in a variety of tissues leads to activation of the hypoxia inducible factor (HIF-1α), which induces transcription of angiogenic genes such as vascular endothelial growth factor (VEGF) [46-49], as well as the MSC chemoattractant stromal cell-derived factor 1 (SDF-1) [50,51]. Once MSC migrate to areas of hypoxia, it has been demonstrated that production of various therapeutic paracrine mediators is increased. Several groups have demonstrated the relevance of hypoxia to MSC growth factor production *in vitro*. For example, exposure of bone marrow (BM)-MSC to 24 hours of hypoxia (1% oxygen) resulted in marked induction of VEGF, Fibroblast growth factor 2 (FGF-2), Hepatocyte growth factor (HGF), and Insuline like growth factor 1 (IGF-1) production, in an NF-kappa β dependent manner [52]. The stimulation of growth factor production by hypoxia is not specific to BM-MSC and has been demonstrated in MSC derived from adipose tissue [53], placenta [54], and dental pulp [55]. Furthermore, hypoxia stimulation of angiogenic and anti-apoptotic factors such as VEGF, FGF-2, HGF and IGF-1 has been reported to also occur in MSC from aged animals, supporting clinical utility [56].

The biological relevance of MSC-secreted growth factors stimulated by hypoxia can be seen in studies showing that conditioned media from MSCs grown under hypoxic but not normoxic conditions endow therapeutic benefit in animal models. For example, Chang *et al*. demonstrated that conditioned medium from hypoxia treated BM-MSC was capable of restoring neurological function in a rat model of traumatic brain injury significantly better than administration of conditioned medium from normoxia conditioned BM-MSC. Furthermore, they demonstrated that efficacy was associated with production of HGF and VEGF, which were involved in the induction of endogenous neurogenesis [57]. In a similar study, the therapeutic activity of hypoxic and normoxic conditioned BM-MSCs was compared in a rat massive hepatectomy model. Hypoxic conditioned BM-MSCs produced significantly higher levels of VEGF *in vitro* as compared to control treated cells. Furthermore, *in vivo* administration resulted in significantly elevated cyclin D1, proliferating cell nuclear antigen-positive hepatocytes, liver weight/body weight ratio, and survival compared with animals that received normoxia preconditioned BM-MSC. Interestingly, blockade of VEGF by *in vivo* administration of anti-VEGF antibody negated the therapeutic effect of hypoxia [58]. In a rat model of diabetic cardiomyopathy it was demonstrated that administration of hypoxia treated BM-MSC resulted in superior inhibition of pathological condition as compared to administration of control BM-MSC. The therapeutic effect was associated with protection of cardiomyocytes by increasing the activity of matrix metalloproteinase-2; inhibiting Transforming growth factor beta 1 (TGF-β1) and caspase-3 and, upregulating Bcl-2/Bax ratio [59].

Hypoxia not only triggers production of growth factors from MSC, but also allows the MSC to retain an undifferentiated phenotype, allowing for self-renewal without differentiation. This may be due in part to the fact that anatomically, MSCs tend to be found in hypoxic areas of the body, i.e. adipose tissue and bone marrow are relatively poorly perfused by the circulatory system [48,60]. It was demonstrated *in vitro* that exposure of BM-MSCs to hypoxia results in augmented cellular proliferation and the formation of colonies in the colony-forming unit assay (CFU-A) and the expression of stemness markers Rex-1 and Oct-4, thereby suggesting an increase in the stemness of BM-MSC when exposed to hypoxia [49].

One of the key factors of MSC of relevance to therapeutics development is their known anti-inflammatory/immune modulatory properties. The potency of this effect is seen in clinical studies showing efficacy of MSC at inhibiting lethal, immune-based condition of graft versus host disease [61-66]. Exposure of MSC to hypoxia has been shown in several systems to augment immune modulatory activity. In one example, MSC expression of the tryptophan catabolizing enzyme indolamine 2,3 deoxygenase (IDO) was markedly upregulated in the presence of hypoxia [67]. IDO is critical in immune regulation by MSC in part through induction of T cell anergy [68], and in part by stimulation of T regulatory cells (T-regs) [69,70]. The practical relevance of hypoxia-stimulated immune regulation of MSCs is seen in the situation of allogeneic use of BM-MSCs for stimulation of therapeutic angiogenesis. It was shown in a recent study that hypoxia-conditioned BM-MSCs from B6 mice ameliorate limb ischaemia of Balb/c mice compared to normoxic MSCs. Histological staining demonstrated that hypoxic BM-MSC have an increased ability to engraft in allogeneic recipients by reducing natural killer cells (NK) cytotoxicity, and decrease the accumulation of host-derived NK cells when transplanted *in vivo*. These allogeneic hypoxia treated BM-MSCs gave rise to CD31+ endothelial cells and αSMA + and desmin + muscle cells, thereby enhancing angiogenesis and restoring muscle structure. Moreover, application of anti-NK antibodies together with normoxic MSCs enhanced angiogenesis and prevented limb amputation in allogeneic recipients with limb ischemia, thus demonstrating that the benefit of hypoxic conditioning was mediated by enhanced immune modulation in the allogenic setting [71].

In short, hypoxic conditioning of cultured MSCs may result in increased production and secretion of trophic factors, augmentation of angiogenic effects, and enhanced immune modulating activity from the conditioned cells relative to normoxic culture conditioning.

#### Inflammatory stimuli

In addition to responding to hypoxia, MSC produce immune modulatory and regenerative factors in response to inflammatory stimuli. One of the most studied mechanisms by which inflammation triggers MSC activity is treatment with interferon gamma (IFN-γ). This cytokine is typically produced during inflammatory Th1 immune responses that are associated with autoimmunity mediated by cellular means, such as CD8 T cells and NK cells. Examples of conditions associated with this type of immune response include multiple sclerosis, diabetes type 1, and rheumatoid arthritis [72]. Exposure of MSC to INF-γ has been demonstrated by numerous groups to increase the immune suppressive activity by stimulation of the enzyme IDO [73-76]. As expected, exposure to this inflammatory mediator induced production of other inhibitors of inflammation by MSCs, including the complement inhibitor Factor H [77], as well as the immune modulatory molecules TGF-β and HGF [78]. At a functional level, Noone *et al*. demonstrated that INF-γ pretreatment of MSC resulted in protection of MSCs from NK-mediated killing in part through upregulation of prostaglandin E (PGE)-2 synthesis [79]. IFN-γ, but also tumor necrosis factor-alpha (TNF-α), IL-1α, and IL-1β induce Gal-9 in MSC [80].

Another inflammatory mediator known to induce regenerative activities in MSC is the macrophage-derived cytokine TNF-α. TNF-α pretreatment of MSCs endowed the cells with superior angiogenic activity *in vitro*, as assessed by expression of VEGF, as well as *in vivo* in an animal model of critical limb ischemia, as compared to untreated MSCs [81]. Another study demonstrated that TNF-α pre-conditioning increased proliferation, mobilization, and osteogenic differentiation of MSCs and up-regulated bone morphogenetic protein-2 (BMP-2) protein level. BMP-2 silencing by siRNA partially inhibited osteogenic differentiation of MSC induced by TNF-α [82]. More recent studies have shown that activators of innate immunity, such as lipopolysaccharide, and toll like receptor (TLR) agonists, also are capable of stimulating regenerative activity of MSCs through induction of production of paracrine factors such as VEGF [83]. IFN-γ and TLR also up-regulate the glucocorticoids production which decreases T-cells stimulated by radiotherapy in colonic mucosa [84]. In general there is evidence to suggest that inflammatory stimuli enhance the regenerative potential and anti-inflammatory response of MSCs.

#### Tri-dimensional culture system activation

MSC are most typically grown *in vitro* in monolayer systems in surface treated plastic flasks. Tridimensional configurations such as spheroid culture have been shown to stimulate higher levels of trophic factor secretion compared to monolayer culture. One of the first observations were that lung MSC micro-emboli of myocardial infarcted mice produced TNF-stimulated gene 6 protein (TSG-6) and found it to be an important anti-inflammatory factor that improved outcomes [42]; TSG-6 is not found in significant quantities in standard MSC monolayer culture. Bartosh *et al*. [85] found that hanging drop induced MSC spheres containing 25 K cell per drop produced significantly higher TSG-6 than monolayer cultures with production increasing over 4 days of culture. In addition, they found higher expression of the anti-inflammatory and antiapoptotic protein STC-1, CXCR4; and three anticancer proteins: TRAIL, IL-24 and CD82 in the MSC spheres.

Conditioned medium (CM) from human MSC spheroids inhibited production of TNF-α, CXCL2, IL6, IL12p40, and IL23 from LPS stimulated macrophages, and presented higher production of prostaglandin E2 (PGE2). This anti-inflammatory and immune modulator is produced through a caspase-dependent IL-1 pathway [86,87]. Of relevancy is that therapeutically, spheroids and spheroid-derived cells were more effective anti-inflammatory agents in a murine model of zymosan-induced peritonitis than monolayer MSC culture cells [85]. Further studies by the same group demonstrated that *in vivo*, intraperitoneal MSCs tended to self-aggregate resulting in self-activation of increased production of trophic factors [87].

Dynamic cultures using spinner flask or rotating wall vessel bioreactor have shown to form small spheroids and demonstrated better osteogenesis and adipogenesis differentiation characteristics, as well as higher concentration of IL-24 [88].

**Figure 1:**
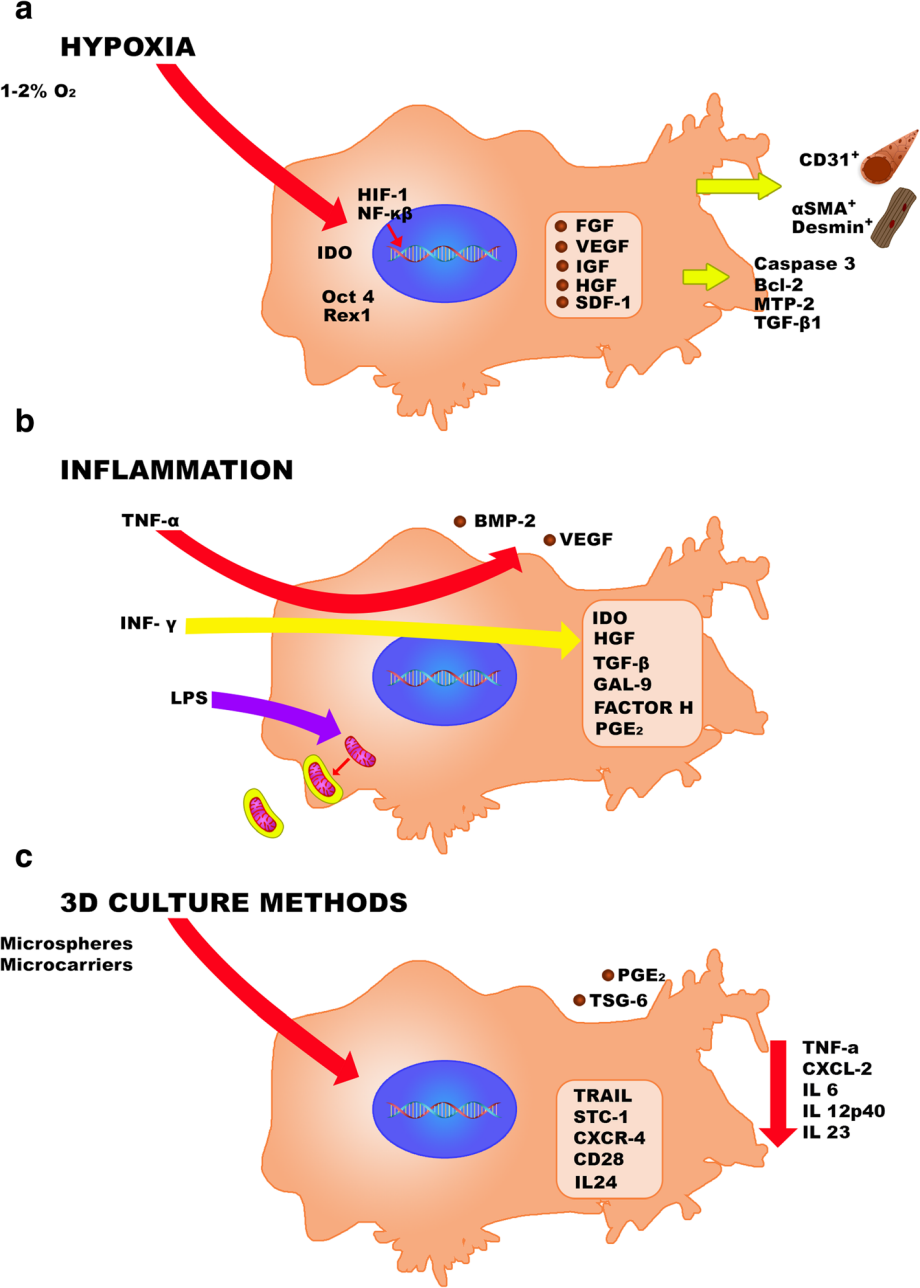
**Effects of the hypoxia**, **inflammation**, **and 3D culture on MSC in terms of expression and secretion of molecules of interest for cell therapy. a)** Hypoxia activates the HIF and the NF-kappa β; increases the expression of several growth factors (inside the square), it also induces IDO activity and enhances stemness (Oct-4 and Rex-1). Also, the hypoxia pre-conditioned MSC, favor the activation of caspase 3, Bcl-2, MTP-2, TGF- β1 on target cells improving apoptosis resistance; improve regenerative capacity of muscle and endothelial cells. **b)** Inflammation induced by INF-γ increases the expression of anti-inflammatory and regenerative molecules (in the square) and, through TNF-α enhances the production of VEGF and BMP-2 which favor formation of new vessels and osteoblasts respectively. Also MSC exposed to LPS are able to encapsulate mitochondria and deliver them to other cells. **c)** 3D culture methods such as microcarriers or spheroids induce the production of TSG-6 and increases PGE_2_ secretion. Besides, it also favor the secretion of antiapoptotic and anticancer molecules (in the square). Further, MSCs obtained from 3D configurations, inhibit the expression of inflammatory and cancer related molecules in target cells.

Overall, these data suggest that the paracrine effects of MSCs are inducible, and have a relationship with context-, or niche-specific settings (Figure 1).

### MSC produced biomolecules

As described above, MSCs produce a plethora of biologically active molecules in response to various stimuli. In this section we will list some of the common molecules associated with MSC activity, and describe their biological significance.

#### Hepatocyte Growth Factor (HGF-1)

Originally discovered as a gene transcript associated with liver regeneration [89], HGF-1 is the high affinity ligand for the receptor tyrosine kinase Met, whose activation is associated with a variety of regenerative activities including angiogenesis, myogenesis, and hematopoiesis [90]. HGF-1 is secreted as a single inactive polypeptide and is cleaved by serine proteases into a 69-kDa alpha-chain and 34-kDa beta-chain. A disulfide bond between the alpha and beta chains produces the active, heterodimeric molecule. The protein belongs to the plasminogen subfamily of S1 peptidases but has no detectable protease activity. Demonstration of regenerative activity by HGF-1 outside of the liver has been shown by experiments demonstrating involvement of this cytokine in acceleration of wound healing, including in cutaneous [91], corneal [92] and gastric [93] wounds. Other biological activities of HGF-1 include stimulation of angiogenesis, which was demonstrated by studies in which injection of HGF-1 plasmid resulted in limb salvage in animals [94], and humans [95,96], in limbs with poor circulation. In line with other soluble factors associated with regenerative processes, HGF-1 possesses immune modulatory activity. Treatment of dendritic cells with HGF-1 results in reduction of ability to induce generation of inflammatory Th1 cells, in part through blocking expression of co-stimulatory molecules such as CD80 and CD86 [97]. Furthermore, studies have shown that *in vivo* administration of HGF-1 protects against autoimmune disease such as experimental autoimmune encephalomyelitis, and collagen induced arthritis, through stimulation of T-regs producing the immune suppressive cytokine IL-10 [98,99].

MSC production of HGF-1 has been shown to be critical in several *in vivo* therapeutic activities of MSCs. One example is in immune modulation associated with intravenous administration of MSCs in models of autoimmunity. Bai *et al*. demonstrated that administration of either MSCs or MSC conditioned medium were capable of suppressing progression, and inducing remission of disease pathology in the EAE model of multiple sclerosis. Serial sections of tissue from treated mice revealed high concentrations of HGF-1, which was also found in the conditioned media. Blocking antibodies to HGF-1 were demonstrated to negate the protective effects of MSCs or MSC conditioned media in this model [100]. Neuroprotective effects of MSC conditioned media also appeared to be dependent on HGF-1, based on experiments in which neutralization of HGF-1 resulted in loss of protection from apoptosis in a glutamate induced excitotoxicity model [101]. Suppression of apoptosis by HGF-1 was also demonstrated to be essential for the therapeutic effects of adipose derived MSCs in a rodent model of acute kidney failure induced by high dose cisplatin. Yasuda *et al*. [102], demonstrated that while local administration of adipose MSCs was capable of reducing acute tubular necrosis and loss of kidney function after cisplatin administration, these effects were negated by administration of anti-HGF-1 antibodies.

Thus the effects of HGF-1 generated by MSCs appear to be multifunctional, directed towards the combination of: angiogenesis; immune modulation; and protection from apoptosis.

#### Transforming Growth Factor Beta (TGF-β)

TGF-β is a protein generally known to possess autocrine inhibitory activities to non-malignant cells, and is widely expressed in a variety of tissues in a latent form [103]. TGF-β signals through the SMAD family of intracellular proteins and its overexpression is associated with fibrotic disease [104]. Local production of TGF-β is a potent mechanism of immune suppression in a variety of contexts including tumor [105,106], pregnancy [107], ocular [108], and testicular immune privilege [109]. Mechanistically, TGF-β acts by suppressing dendritic cell maturation [110], stimulating T-regs production [111], and suppressing generation of inflammatory Th17 cells [112].

MSC production of TGF-β has been demonstrated in MSCs derived from numerous tissues including adipose [113], bone marrow [114], and umbilical cord [115]. The essential contribution of TGF-β to suppression of T cell proliferation by MSC was originally demonstrated by Zhao *et al*. [116]. They showed that antibody neutralization resulted in restoration of lymphocyte proliferation. Subsequent studies have demonstrated that MSC administration to animals suffering ischemic injury to the CNS resulted in improved neurological outcomes, which was abrogated by silencing TGF-β in the administered MSC [117]. The TGF-β dependent therapeutic effects were associated with reduction of inflammatory cytokine expression in the CNS and suppression of microglial activation.

MSC-based immune modulation has been shown by several investigators to be mediated in part by TGF-β. Ye *et al*. utilized an *in vitro* model of T-regs generation to demonstrate that MSC-derived TGF-b is necessary for differentiation of FoxP3 expressing T-regs from naïve T cells in an antigen-nonspecific system [118]. In an *in vivo* study, administration of MSC into a bacterially-induced hepatic injury model was shown to result in alleviation of hepatotoxicity, mediated in part by TGF-β dependent generation of T-regs [118].

Overall, the effects of TGF-β production by MSC appear to relate primarily to immune modulation. Given the profibrotic role of TGF-β in various pathological conditions, the balancing act that this cytokine plays in the mediation of MSC derived therapeutic activities is subject of intense investigation.

#### Vascular Endothelial Growth Factor (VEGF)

VEGF was one of the first described soluble angiogenesis stimulation factors [119]. This naturally occurring glycoprotein, which acts as a growth factor for endothelial cells, is produced by a variety of tissues in response to reduced oxygen tension, whose transcription is mediated in part by activation of the HIF-1 [120]. The essential role of VEGF in angiogenesis is exemplified by numerous studies demonstrating that blockade of this protein is therapeutic in angiogenesis-mediated diseases such as neoplasia and wet macular degeneration [121,122]. Conversely, administration of VEGF protein or DNA plasmid induces angiogenesis in animal models and in clinical trials [123,124]. Unfortunately, clinical use of VEGF for treatment of ischemic conditions has not met the required endpoints in Phase III clinical trials, in part due to poor regulation of VEGF-induced angiogenesis. In contrast, MSC expression of VEGF appears to be tightly regulated based on physiological need, and may represent a superior means of inducing therapeutic angiogenesis [125].

The role of VEGF in MSC-mediated angiogenesis was initially described in studies of bone marrow MSC administration into ischemic myocardium in animal models of heart failure. Stimulation of angiogenesis and endothelial cell proliferation was associated with MSC expression of VEGF in both small and large animal studies [126,127]. The superior effect of MSC administration in heart failure models, compared to administration of VEGF alone was demonstrated in subsequent studies [128]. Suggesting causative effects of VEGF in angiogenesis are studies showing that blockade of VEGF blocks MSC-induced angiogenesis in several animal models [129,130]. Furthermore, VEGF works as anti-apoptotic molecule suppressing p53-mediated apoptosis by activation of FAK (focal adhesion kinase), and also by promoting Bcl-2 and A1 [131,132]; clinical response to cell-based intervention has been associated with increases in serum VEGF levels [133].

#### Tumor necrosis factor-stimulated gene-6 (TSG-6)

TSG-6 is a 35 kDa glycoprotein which was originally discovered in the synovial fluids of patients with arthritis and serum of patients with inflammatory diseases [134]. Physiologically, it appears that one of the functions of TSG-6 is to counteract inflammatory effects of TNF-a and IL-1 [135]. Relating to MSC, original studies by Prockop *et al*. examined soluble mediators that may be responsible for regenerative effects of intravenously administered MSC in an animal infarct model. Cell tracking studies revealed that the majority of administered MSC lodged into the lung, however, potent post-infarct regeneration was observed. Using microarray analysis, it was found that TSG-6 was one of the most highly up-regulated transcripts in lung-lodged MSC. Interestingly, silencing of TSG-6 in the administered MSC resulted in loss of therapeutic activity, whereas, administration of exogenous TSG-6 resulted in replication of therapeutic activity [42]. MSC therapeutic activities in other animal models of disease was observed to be dependent on TSG-6, including cerebral ischemia [136], diabetes type 1 [137], peritoneal adhesions [138,139], and experimental autoimmune encephalomyelitis (EAE) [140].

#### Prostaglandin E2 (PGE2)

PGE2 is a 352 Da molecule belongs to the prostanoid family of small molecules, and is a product of arachidonic acid metabolism by the cyclo-oxygenase family of enzymes. Immune suppressive activities of PGE2 have been well characterized by type 2 macrophages and immature dendritic cells as a means of feedback inhibition after immune activation [141]. Inhibition of T cell activation by myeloid suppressor cells is also induced by PGE2 in models of cancer and pregnancy [142-145]. Mechanistically, PGE-2 inhibits various immune cells including NK cells [146], granulocytes [147], dendritic cells [148], and Th1 cells [149]. Additionally, PGE2 has also been shown to directly induce differentiation of T-regs expressing FoxP3 from naive T cells [150].

One of the most potent demonstrations of the ability of MSC-derived PGE2 to alter disease pathology was a study by Nemeth *et al*., in which BM-MSC inhibited sepsis in the aggressive cecal-puncture ligation model. Protection from sepsis was associated with generation of IL-10 producing macrophages, whose differentiation was dependent on MSC-produced PGE-2 [151]. Subsequent studies have supported the pivotal role of MSC generated PGE-2 in mediation of anti-inflammatory activities. Zhang *et al*. demonstrated that MSC administration into a bacterial-induced model of liver failure resulted in protection from lethality. Protection was associated with generation of T-regs and increases in serum IL-10. The systemic administration of COX inhibitors abrogated the protective effect [152]. In models of T cell mediated immune pathology, MSC administration inhibited allogeneic cardiac allograft rejection. When blockade of PGE2 generation was accomplished by COX inhibition, graft rejection, mediated by Th1 cells was observed [153].

PGE2 thus appears to be one of the major mediators of MSC associated immune modulation, specifically by acting as a promoter of T-regs and inhibitor of inflammatory responses. This is somewhat paradoxical to observations that systemic administration of PGE2 at large concentrations is pro-inflammatory. These differences are explained in part by various affinity PGE2 receptors on target tissues, and illustrate the biological complexity of MSC activities.

PGE2 is produced in different concentrations by MSCs depending on the source. Amniotic membrane [154] and chorionic villi [12] MSC produced higher concentrations of PGE2 under *in vitro* culture conditions than bone marrow or cord derived MSC. This variable should be considered if beneficial effect of PGE2 is desired for clinical applications.

#### Galectin 1 and 9

Galectins are a family of proteins that share characteristic amino acid sequences and affinity for β-galactoside sugars, such as N-acetyllactosamine (Galβ1-3GlcNAc or Galβ1-4GlcNAc), and have consecutive numbers [155]. Gieseke group [156] have shown that Galectin 1 plays an important role in the immonumodulatory capacity of MSC. They demonstrated that T cells regulation capacity was diminished significantly in galectin-1 knockdown cells compared to wild type because of partially restored proliferation of CD4^+^ and CD8^+^ T cells and that the release of TNFα, IFNγ, IL-2 and IL-10 was modulated by galectin 1.

MSC immunomodulation is also affected by galectin-9, which is highly induced by inflammatory stimuli intracellular and also in the conditioned medium. Galectin-9 knockdown cells lose an important portion of their T-Cell antiproliferative effect [80].

#### MSC derived microvesicles

Production of microvesicles by MSC has been reported to be associated with regenerative activities. Microvesicles are generated from budding of the cell membrane and are considered to be 50 nm – 1000 μm in size. One of the first descriptions of microvesicles as related to regenerative medicine was by Quesenberry’s group who demonstrated that culture of injured adult tissue with bone marrow cells results in bone marrow differentiation into cells of similar lineage as the injured tissue, with the proclivity of differentiation being mediated by microvesicles released from the injured tissue [157]. Subsequent studies have shown that there is a bidirectional communication between injured tissue and cells with regenerative potential, in that various stem cells also release microvesicles [158]. Specific examples of the regenerative potential of microvesicles follow. Bruno *et al*. utilized a glycerol-induced SCID mouse model of acute kidney injury, in which human MSC derived microvesicles were administered intravenously. Protection from acute kidney injury was observed, which was correlated with transfer of human miRNA into tubular epithelial cells. Treatment of microvesicles with RNAse resulted in abrogation of protective effects. This study supported the concept that MSC exert a protective effect against cellular apoptosis through transfer of miRNA [159]. In another model of kidney injury, Gatti *et al*. administered human MSC derived microvesicles into a renal ischemia reperfusion model [160]. A dose-dependent inhibition of acute tubular necrosis was observed, which correlated with preservation of renal function. Similar to the previously described study, protection from kidney injury was dependent on functional miRNA since treatment with RNAse eliminated protective activity.

Previous studies have suggested that MSC conditioned media is capable of stimulation proliferation of endothelial cells in vitro, and angiogenesis in vivo. Although the involvement of angiogenic cytokines such as VEGF and HGF-1 was previously believed to be responsible for this effect, antibody-blocking was not able to achieve 100% inhibition of angiogenesis. Zhang *et al*. studied microvesicles collected from hypoxia preconditioned MSC CM, and demonstrated that microvesicles can be internalized by umbilical cord endothelial cells and promote proliferation in a dose-dependent manner. Also, MSC microvesicles stimulate angiogenesis in a hind limb ischemia model [161].

Another therapeutic activity of microvesicles appears to be related to suppression of alveolar inflammation in models of acute lung injury. Previous studies have demonstrated that intravenous MSC administration protects animals from endotoxin induced lung injury). Zhu *et al*. showed a dose-dependent reduction in water leakage and neutrophilic infiltration into the lung when intrapulmonary administration of bone marrow derived MSC isolated microvesicles were used in a similar model [162]. Interestingly, neutralization of therapeutic activity was observed by blockade of keratinocyte growth factor using siRNA.

Another important study by Islam *et al*. shows the capacity of MSC to deliver encapsulated mitochondria in lung epithelial cells treated with LPS, leading to the survival of the host cells [163].

Overall, these studies suggest that microvesicles play an important role in MSC associated paracrine therapeutic activities, which may complement activities associated with release of soluble proteins and small molecules by MSC.

#### MSC derived exosomes

Exosomes are nanoparticles (40-100 nm) in size that possess highly defined homogeneous characteristics [164]. Originally, thought to be a by-product of cell protein turnover [165], these nanoparticles are becoming appreciated as a critical means of intracellular communication in areas ranging from neurotransmission [166], to immune modulation [165], to infectious disease [167].

Compared with other secreted vesicles such as microvesicles (described above), exosomes have much better defined biophysical and biochemical properties, specifically, they have a diameter of 40–100 nm (with a density in sucrose of 1.13–1.19 g/ml, and can be sedimented at 100,000 g [164]. Their membranes are enriched in cholesterol, sphingomyelin and ceramide, and are known to contain lipid rafts. Exosomes were originally discovered as a means of exportation of the transferrin receptor during sheep reticulocyte maturation [168]. In recent years an explosion of interest in exosomes has occurred, with a wide variety of cells being reported to secrete these nanoparticles ranging from T cells [169,170], B cells [171,172], dendritic cells [173,174], tumor cells [175,176], neurons [177,178], oligodendrocytes [179], and placental cells [180]. Additionally, there is evidence that immune escape of the “fetal allograft” is associated with exosomes [181].

While MSC have been previously demonstrated to exert therapeutic effects in animal models of cardiac infarction, Lai *et al*. asked whether MSC-derived exosomes exert similar effects. They reported that MSC cultures generate phospholipid containing vesicles consisting of cholesterol, sphingomyelin, and phosphatidylcholine. These vesicles were believed to be exosomes based on coimmunoprecipitating with exosome-associated proteins, such as CD81, CD9, and Alix. These particles were purified as a homogeneous population of particles with a hydrodynamic radius of 55–65 nm by size-exclusion fractionation on a HPLC. It was found that administration of these particles, which resembled exosomes biochemically, to animal cardiac infarct models resulted in reduction of infarct size and improved heart function [162]. Mechanistically, the effects of MSC-derived exosomes on cardiac infarct appear to be mediated by increasing levels of ATP and NADH in cardiomyocytes, as well as decreasing oxidative stress and increased phosphorylated-Akt and phosphorylated-GSK-3β [182].

In addition to protection from ischemia reperfusion injury, MSC functions such as inhibition of fibrotic injury have also been shown to be mediated by MSC-derived exosomes. Li *et al*. utilized the carbon tetrachloride model of fibrotic liver injury to demonstrate that administration of MSC-derived exosomes inhibited collagen deposition and preserved liver function in a manner similar to administration of MSCs themselves [183].

Mechanistically, the regenerative activities of MSC-derived exosomes are a subject of ongoing investigation. Some studies suggest miRNA transfer by MSC-derived exosomes mediates various therapeutic effects [184], in a manner similar to microvesicles. Other studies suggest the involvement of exosome associated proteins such as lactadherin, or galectins, which are known to possess anti-inflammatory functions [185].

## Conclusions

MSCs are rapidly emerging as a clinically-viable cell therapy, with numerous trials ongoing, and registration for marketing approval in several jurisdictions. The paradigm shift that MSCs activities are mediated by secreted factors as opposed to the previous notion of differentiation into injured tissue offers numerous possibilities for therapeutic development based on MSC secreted products. Current investigations at replicating *in vitro* the optimal environment for MSC production of therapeutic factors will lead to development of therapies utilizing MSC secreted factors which will alleviate the need for administration of cells.
